# BZW2 gene knockdown induces cell growth inhibition, G1 arrest and apoptosis in muscle‐invasive bladder cancers: A microarray pathway analysis

**DOI:** 10.1111/jcmm.14266

**Published:** 2019-04-01

**Authors:** Haifeng Gao, Guanghai Yu, Xian Zhang, Song Yu, Yu Sun, Yinghua Li

**Affiliations:** ^1^ Department of Urology Surgery Dalian Municipal Central Hospital Dalian China; ^2^ Department of Oncology The Second Affiliated Hospital of Dalian Medical University Dalian China

**Keywords:** BZW2, microarray pathway analysis, muscle‐invasive bladder cancers (MIBCs), xenograft model

## Abstract

Bladder cancer is among the most common cancers all over the world. The function of basic leucine zipper and W2 domains 2 (BZW2) in tumour progression has been reported. However, the biological function of BZW2 in muscle‐invasive bladder cancers (MIBCs) remains to be determined. The aim of the present study was to reveal the expression and roles of BZW2 in human MIBCs and to explore the molecular mechanisms underlying these functions. Clinically, BZW2 expression was higher in MIBC tissues than the adjacent non‐tumour tissues. Knocking down BZW2 using shRNA inhibited cell proliferation and G1/S cell cycle progression in vitro, and induced apoptosis in both 5637 and T24 cells. Moreover, in vivo studies with mice xenograft models confirmed the anti‐proliferative effects of BZW2‐knockdown, providing a future therapeutic target. We also performed biochemical microarray analysis to identify the potential signalling pathways, disease states and functions which could be affected by suppressing BZW2 in MIBC cells. Collectively, our findings suggest BZW2 has an oncogenic role in MIBCs and serves as a promising target for molecular diagnosis and gene therapy.

## 1 INTRODUCTION

Bladder cancer is among the most common cancers all over the world, with approximately 380,000 new cases and 150,000 deaths per year.^1^ It ranks fifth among cancers in men in western countries.^2^ Age is the most significant risk factor for bladder cancer, and median age at diagnosis is about 70 years.^3^ Bladder cancer poses a considerable economic burden primarily owing to the lifetime surveillance and repeated treatment of recurrent disease.^4^ According to the extent of invasion, it consists of muscle‐invasive bladder cancers (MIBCs) and non‐muscle‐invasive bladder cancers (NMIBCs). Although only 20% of bladder cancer patients are diagnosed with MIBCs, the vast majority of cancer‐specific deaths are attributed to MIBCs.^5^ Even worse, MIBCs have less favourable prognosis and common progression to metastasis although the treatment has not advanced for several decades.^2^ Therefore, new approaches to systemic therapy are definitely needed.^6^

Whole‐genome analyses have revealed that MIBCs are heterogeneous.^7^ A wide variety of oncogenes were found to be altered in bladder cancer, including genes associated with protein tyrosine kinase signalling, cell cycle regulation and others.^8^ Among them, aberrations in cell‐cycle regulation are one of the most extensively studied molecular aspects of bladder cancer.^9^ For instances, increasing cyclin D1 positivity is regarded as a predictor of improved survival and of a lower progression rate in MIBCs.^10^ Moreover, almost all MIBCs have defects in genes encoding proteins that control the G1 cell cycle checkpoint.^9^ However, there is still no molecular biomarker to predict the progression of disease accurately. Therefore, it demands more efforts to explore the new molecular targets and underlying mechanism for bladder cancer, especially MIBCs.

**Figure 1:**
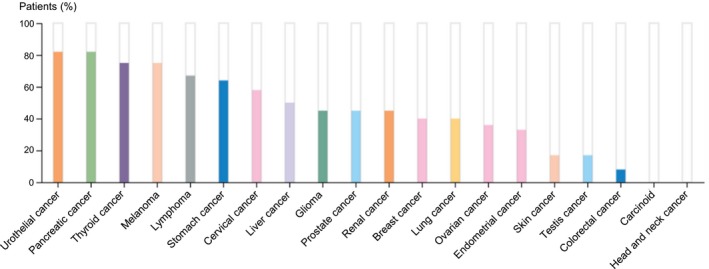
(A) statistical analysis conducted on clinical patients (https://www.proteinatlas.org/ENSG00000136261-BZW2/pathology).^14^, ^15^, ^16^ For each cancer, colour‐coded bars indicate the percentage of patients with high and medium protein expression level. The cancer types are colour‐coded according to which type of normal organ the cancer originates from. Low or not detected protein expression results in a white bar.

Basic leucine zipper and W2 domains 2 (BZW2) is a member of the bZIP superfamily of transcription factors.^11^ BZW2 is an evolutionary highly conserved protein and involved in cell‐cell adhesion via cadherin binding.^12^ BZW1, another member of the bZIP superfamily, has been recognized as a novel proliferation regulator in salivary mucoepidermoid carcinoma.^13^ In contrast, there was little study reported on the potential role of BZW2 in cancers. Most recently, Cheng et al reported that BZW2 is up‐regulated in osteosarcoma and its down‐regulation inhibits cell growth by inactivating the Akt/mTOR signalling pathway,^11^ suggesting BZW2 plays a potentially important role in osteosarcoma progression. In addition, a statistical analysis conducted on clinical patients (https://www.proteinatlas.org/ENSG00000136261-BZW2/pathology) 14, 15, 16 showed that high expression of BZW2 is most common in urothelial cancer among a wide variety of different cancers (Figure 1). Nonetheless, it remains unclear about the exact role of BZW2 in context of MIBCs.

In the present study, we combined in vitro, in vivo, bioinformatics and clinical studies to explore the function and possible mechanism of BZW2 in MIBCs. We evaluated the expression level of BZW2 in clinical patients with advanced bladder cancer (of stage T2 and above), as well as in two different human MIBC cell lines (5637 and T24). We also assessed the effects of BZW2 knockdown on cell growth, cell cycle progression, cell death in vitro, as well as the tumour growth inhibition in vivo. The signalling pathways and disease states affected by BZW2 knockdown were further analysed, which could provide insights into the possible mechanism behind the BZW2 function in MIBCs.

## 2 MATERIALS AND METHODS

### 2.1 Cell line and cell culture

Human MIBC cell lines (T24 and 5637) and normal bladder epithelial cell line (SV‐HUC‐1) were purchased from the Cell Bank of Shanghai Institutes for Biological Sciences (Chinese Academy of Sciences, Shanghai, China) and cultured in RPM1 1640 medium (Hyclone, Logan, UT) containing 10% fetal bovine serum (FBS) (Thermo Fisher Scientific, Inc, Rochester, NY) and 1% penicillin/streptomycin solution (Solarbio, Shanghai, China) at 37°C. The MIBC tissues and adjacent non‐tumour para‐carcinoma tissues were obtained from a representative patient with MIBC.

### 2.2 Animals

Male BALB/c Nude mice (6‐8 weeks age) were obtained from the Animal Center of Dalian Medical University (Dalian, China). Protocols involving the use of the animals were approved by the Dalian Medical University Animal Policy. All animal care and experiments were performed in accordance with the approved protocols by the Animal Studies Subcommittee and the ‘The Detailed Rules and Regulations of Medical Animal Experiments Administration and Implementation’ (Document No. 1998‐55, Ministry of Public Health, China).In addition, the animal laboratory recieved animal use and production certificate issued by the Science & Technology department of Shanghai (Certificate number: SYXK2015‐0018 and SCXK2013‐0018).

### 2.3 Plasmids construct and gene knockdown

The shRNA sequence corresponds to Homo sapiens basic leucine zipper and W2 domains 2 (BZW2), (GenBank accession number NM_014038). Duplex shRNA oligos were cloned into the AgeI and EcoRI sites in GV115. The sense shRNA strand of BZW2 was CCGGGCTGATGTTCTGAGCGAAGAACTCGAGTTCTTCGCTCAGAACATCAGCTTTTTG. The insert fidelity was confirmed by sequencing both strands with the following primers (forward: CCTATTTCCCATGATTCCTTCATA; reverse: GTAATACGGTTATCCACGCG). BZW2 was knocked down with a GV115 based lentiviral vector. Lentiviruses were generated in HEK293T cells as described,^17^ and then cells were infected with virus‐containing supernatants. The alteration of expression of BZW2 was confirmed by real‐time RT‐PCR and Western blotting.

### 2.4 Cell proliferation assay

Cell proliferation ability was measured with 3‐(4,5‐dimethyl‐2‐thiazolyl)‐2,5‐diphenyl‐2‐*H*‐tetrazolium bromide, Thiazolyl Blue Tetrazolium Bromide (MTT) colorimetric assay as described before.^18^ Briefly, cells were seeded into 96‐well plates (2 × 10^3^ cells/well), and cell proliferation was documented every 24 hours. The number of viable cells was assessed by measurement of the absorbance at 490 nm using a DNM‐9602 microplate reader (Pulang New Technology Corp, Beijing, China).

### 2.5 Cell scratch test

The 5637 cells in logarithmic growth phase were treated with serum‐free treatment for 24 hours, adjusted to a cell concentration of 5 × 10^5^ mL^−1^ with RPMI‐1640 medium containing 10% FBS, inoculated into 6‐well plates and incubated in a 37°C, 5% CO_2_ incubator. When the 5637 cells were grown to 90% confluence, the 6‐well plate was removed for scratching. During the scratching process, a 10 μL tip was used to draw straight along the marking line, and the floating cells were washed away with PBS, and then the tumour cells were separately added to the medium of BZW2‐knockdown (KD) group and normal control (NC) group. The incubation was continued in an incubator at 37°C in a 5% CO_2_ atmosphere, after 24 hours, the degree of cell migration was observed under a fluorescence microscope. Three replicate wells were set per cell and each experiment was repeated three times.

### 2.6 Cell cycle analysis

The effect of BZW2 knockdown on the cell cycle distribution was measured with PI staining according to previous publication.^19^ After infection with shRNA, about 1 × 10^6^ 5637 or T24 cells were collected, washed with PBS and fixed with 70% ethanol at −20°C overnight. After centrifugation, cells were then resuspended in 1 ml staining solution which contains 50 µg/ml PI (Sigma, St. Louis, MO) and 20 µg/ml RNase A (Fermentas, St. Leon‐Rot, Germany). After incubation of 30 min at room temperature in the dark, samples were analysed with a flow cytometer. The percentage of cells in various phases of the cell cycle was determined using ModFit software.

### 2.7 Apoptosis assay

Apoptotic cells were assessed by staining with Annexin V‐APC Apoptosis Detection Kit (eBioscience, San Diego, CA) following the instructions of standard manual. Specifically, 5 × 10^5^ cells were collected, washed and resuspended in 200 μL Annexin V‐APC binding buffer. After that cells were incubated with Annexin V‐APC (10 μL) in the dark for 15 min, and then supplemented with 600 μL Annexin V‐APC binding buffer. Apoptosis was detected using Guava easyCyte HT flow cytometer (Millipore, Bedford, MD).

### 2.8 Western blot analysis

Western blotting was performed as described before.^20^ Briefly, cells were harvested and lysed after treatment, next the supernatants were collected by centrifugation at 12000 rmp at 4°C. Denatured and fractionated by 12.5% SDS/PAGE, the proteins were transferred to PVDF membranes and probed with anti‐BZW2 (Sigma, St. Louis, MO) (1:300). GAPDH was used as an internal control with anti‐GAPDH (Santa Cruz Biotechnology Inc, Santa Cruz, CA) (1:2000). The X‐ray film was developed by an enhanced chemo‐luminescence system.

### 2.9 RNA isolation and quantitative real‐time PCR

Total RNA was extracted from cultured cells with TRIzol reagents according to the standard manual. RNA was reversely transcribed using RevertAid First Strand cDNA Synthesis Kit before quantification with spectrophotometry. Quantitative real‐time PCR (qRT‐PCR) was carried out on the Applied Biosystems Prism 7300 sequence detection system with Maxima SYBR Green/ROX qPCR Master Mix according to the manual. GAPDH was considered as an internal control. The specific primers used in real‐time PCR were shown as below (Table 1).

**Table 1:** Real‐Time PCR primer sequences

Gene	Accession number	Primer sequence
BZW2	NM_014038	F: 5′‐TTTCTGGACTCTACAGGCTCAA‐3′ R: 5′‐ACCATCATCTATGCGCGTTCC‐3′
GAPDH	NM_001256799.1	F: 5′‐TGACTTCAACAGCGACACCCA‐3′ R: 5′‐CACCCTGTTGCTGTAGCCAAA‐3′

### 2.10 Xenograft mouse model

T24 cells infected with the shRNA lentiviruses (10 h) for the BZW2 genes (KD) and vector control (NC) (GV115‐Green fluorescent protein, GV115‐GFP) were subcutaneously injected into Nude BALB/c (4 × 10^6^/mouse), which were then sorted into two different groups. The amount of cells used in both casees (NC and KD) were 1.00E+07, and the injection site was right subcutaneous axilla. After 6 weeks, bioluminescent signals of tumours in live mice were captured on a Xenogen IVIS Spectrum. Region of interest (ROI) analysis was used to measure light emitted using AMIVIEW software.^21^

### 2.11 Microarray pathway analysis using ingenuity pathway analysis (IPA)

GeneChip PrimeView Human Gene Expression Arrays (Affymetrix UK Ltd., High Wycombe, UK) was applied to study the alternations of canonical signalling pathways in response to BZW2 gene knockdown (KD) in human MIBC cells (T24 cell line). The relative activity of each pathway was determined and normalized to that of normal controls (NC). Expression data were normalized by quantile normalization. All gene level files were imported into Ingenuity Pathway Analysis (IPA) software (Qiagen GmbH, Hilden, Germany)^22^ for further analysis according to previous report^23^. Specifically, the distributions of the intensities of six samples and the similarities between BZW2‐knockdown (KD) and normal control (NC) groups were examined by principal component analysis (Figure S1) and Pearson's correlation of the signal value (Figure S2). Signal value distribution (Figure S3) and relative signal box plot graphs (Figure S4) demonstrated the expression values of all microarray probe distribution statistics and all samples in current study were reproducible. Scatter‐plot graphs (Figure S5) and a dendrogram (Figure S6) demonstrated differences in gene expression of all microarray probe distribution statistics between the KD and the NC group. Differentially expressed genes between six samples were identified through fold change filtering.

### 2.12 Statistical analysis

All experiments were performed in triplicate and the results are expressed as the mean ± SD. Data were analysed using SPSS software (Chicago, IL, USA). Differences with *P*‐values < 0.05 were considered statistically significant.

## 3 RESULTS

### 3.1 BZW2 is overexpressed in MIBC cell lines and MIBC tissues

**Figure 2:**
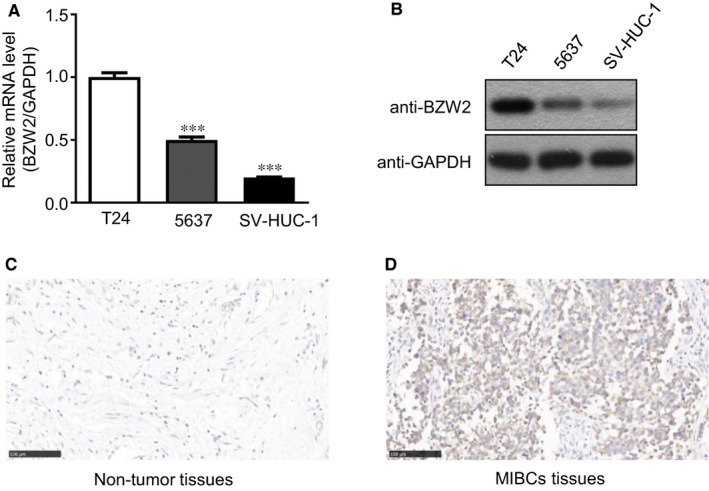
(A), The mRNA expression of BZW2 in 5637, T24 and SV‐HUC‐1 cell lines. B, The protein expression of BZW2 in 5637, T24 and SV‐HUC‐1 cell lines. C, The expression of BZW2 in adjacent non‐tumour tissues of representative patient with MIBC. D, The expression of BZW2 in MIBC tissues of the same patient.

Basic leucine zipper and W2 domains 2 (BZW2) has been recognized to exhibit oncogenic function in osteosarcoma.^11^ With great interest in exploring the potential role of BZW2 in MIBCs, we detected the mRNA expression of BZW2 in human MIBC cell lines (5637 and T24), and SV‐HUC‐1 cell line, with qRT‐PCR, which showed that BZW2 mRNA levels were significantly higher in the MIBC cell lines compared to the SV‐HUC‐1 cell line (Figure 2A). In addition, we verified the above results at the protein level by Western blot analysis (Figure 2B). We also collected clinical tissues to assess the difference of BZW2 expression. From our results, it was shown that BZW2 expression was significantly up‐regulated in MIBC tissues compared with the adjacent non‐tumoUr tissues from the same patient (Figure 2C,D). These results clearly suggest that BZW2 could play an important role in the tumorigenesis of MIBCs. Therefore the rationale for further investigations was validated.

### 3.2 Knockdown of BZW2 inhibits cell growth and migration, and decreased the viable cell number in 5637 and T24 cells

**Figure 3:**
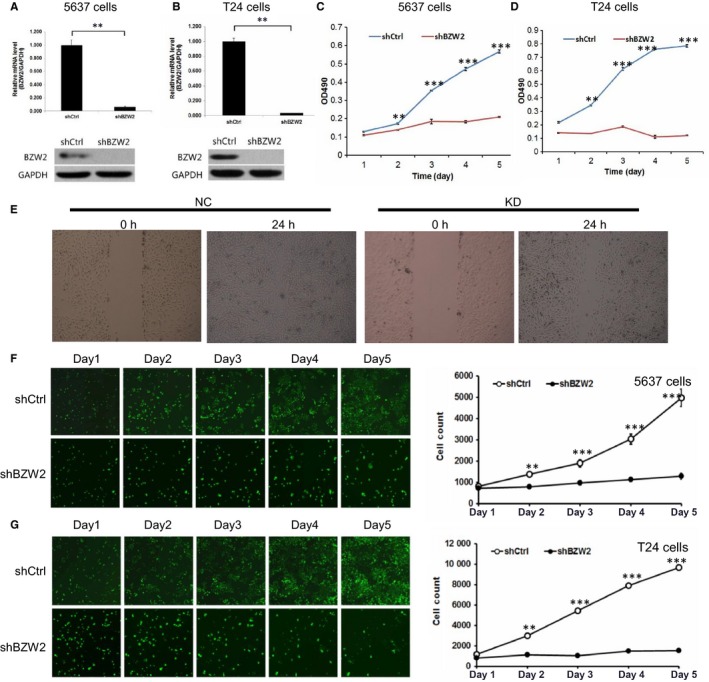
(A), The effects of BZW2 gene knockdown on the mRNA and protein expressions in 5637 cells. B, The effects of BZW2 gene knockdown on the mRNA and protein expressions in T24 cells. C, The effects of BZW2 gene knockdown on the cell growth of 5637 cells. D, The effects of BZW2 gene knockdown on the cell growth of T24 cells. E, The effects of BZW2 gene knockdown on the cell migration of 5637 cells. F, 5673 and (G) T24 cells survival was measured by the Celigo cytometer ex (**P < 0.01, ***P < 0.001)

To explore the functions of BZW2 in MIBCs, gene knockdown with shRNA was used against BZW2 in both human MIBC cell lines (5637 and T24). Prior to the evaluation, we first validated the effect of BZW2‐knockdown by determining the expressions at both mRNA and protein levels with qRT‐PCR and Western blotting, respectively. As shown in Figure 3A,B, it was clear that both mRNA and protein expressions of BZW2 were significantly reduced after the transfection with BZW2‐specific shRNA in 5637 and T24 cells.

On this basis, we detected the effects of BZW2‐knockdown on the cell growth and survival of 5637 and T24 cells. From MTT results, a 5‐day growth curve analysis indicated that the knockdown of BZW2 leads to the dramatic inhibition of cell growth in both MIBC cell lines (Figure 3C,D). Specifically, BZW2‐knockdown reduced the cell growth by approximately 60% and 80% in 5637 and T24 cells separately. The differences observed were statistically significant (*P* < 0.001). In addition, based on the results of the scratch test, we found that the cell migration rate of the KD group was significantly lower than that in the control group after 24 hours, indicating that BZW2 knockdown inhibited the migration of 5637 cells (Figure 3E).

We also implemented Celigo cytometer to measure the viable cell numbers at a variety of time points (eg 1, 2, 3, 4 and 5‐day). We directly counted the cells with bright‐field analysis using the Celigo cytometer (Cyntellect Inc, San Diego, CA). From our results, it can be seen that in both MIBC cell lines (5637 and T24), BZW2‐knockdown significantly decreased the viable cell number compared with the vector control treatment (Figure 3F,G). Collectively, these results suggest an oncogenic role of BZW2 in context of MIBCs.

### 3.3 BZW2 knockdown induces G1‐phase cell cycle arrest and apoptosis in 5637 and T24 cells

**Figure 4:**
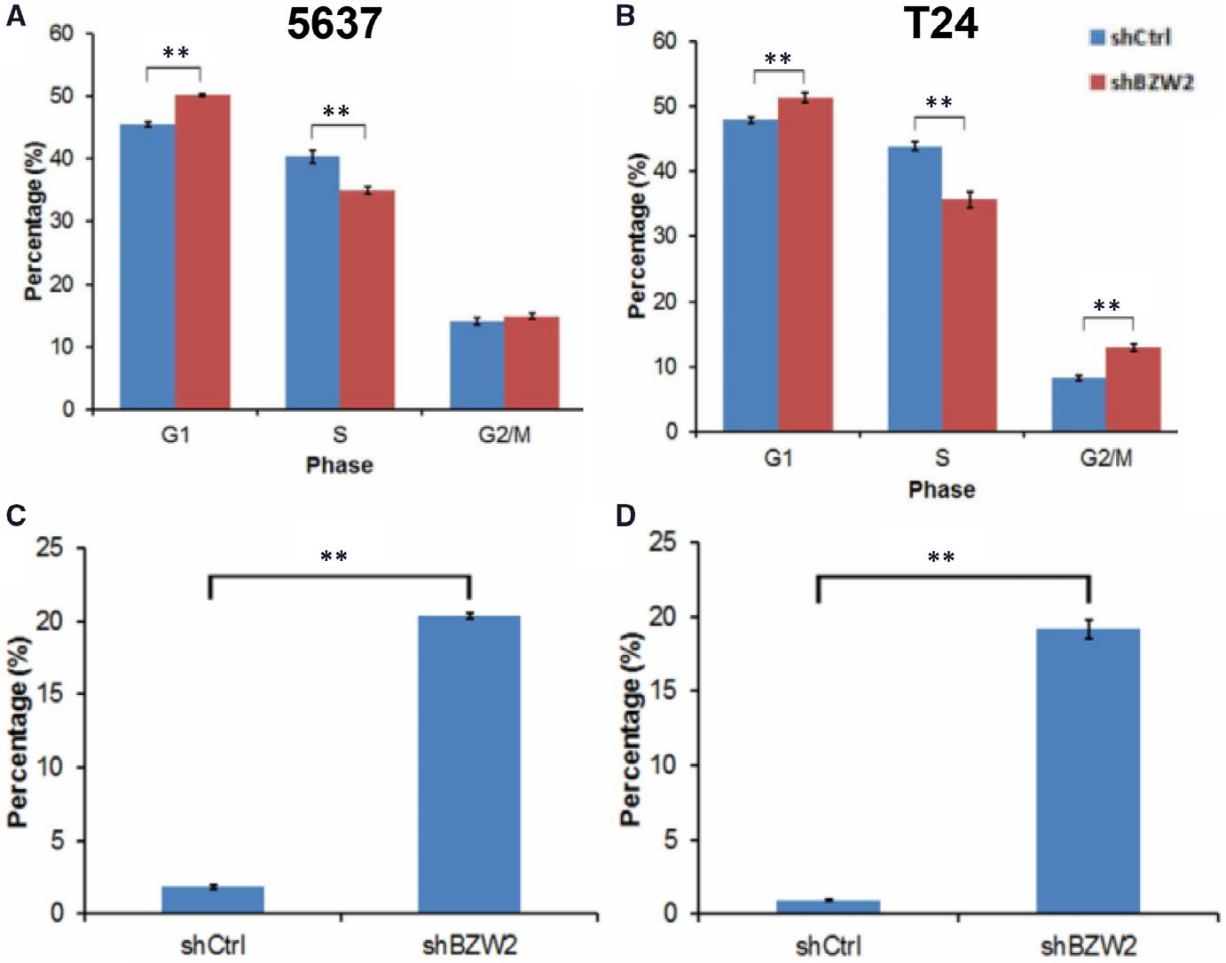
(A), The cell cycle distributions of both BZW2‐knockdown and vector control 5637 cells. B, The cell cycle distributions of both BZW2‐knockdown and vector control T24 cells. C, The percentage of apoptotic cells induced by BZW2‐knockdown and vector control in 5637 cells. D, The percentage of apoptotic cells induced by BZW2‐knockdown and vector control in T24 cells. Asterisks indicate significant difference (**: *P* < 0.01)

We assessed the changes in cell cycle progression in response to BZW2 knockdown with flow cytometry. From our results, it was shown that BZW2‐knockdown induced more accumulation of cells in G1 phase compared with the vector control cells, which could be evidenced by the significant increase of G1‐phase cell percentage (Figure 4A,B). Consistently, this was accompanied by the dramatical reduction of the percentage of cells in S‐phase. Meanwhile, there is no significant difference in the G2/M cell populations between BZW2‐knockdown and vector control cells for 5637 cell line. It was also noted that different from 5637 cells, accumulation of cells in G2/M phase was also observed in BZW2‐knockdown T24 cells, which would be discussed in the following content with assistance of microarray analysis. In conclusion, our data revealed that BZW2 knockdown is able to inhibit the G1‐to‐S cell cycle progression in 5637 and T24 cells.

The decreased viable cell number in conjunction with the induced G1‐phase cell cycle arrest strongly suggested that the cells treated with shRNA of BZW2 undergo apoptosis. To confirm this, we performed annexin V staining and fluorescence‐activated cell sorting analysis. Our results indicated more than 18% increase in the fraction of annexin V positive cells in both BZW2‐knockdown 5637 and T24 cells (Figure 4C,D), confirming the fact that BZW2 knockdown induced significant apoptosis in MIBCs cell line. Together, our findings indicate that the knockdown of BZW2 inhibits cell growth and decreases the viable cell numbers in 5637 and T24 cells by inducing G1‐phase cell cycle arrest and apoptosis.

### 3.4 In vivo study of anti‐proliferative effects of BZW2 knockdown on MIBC xenograft models

**Figure 5:**
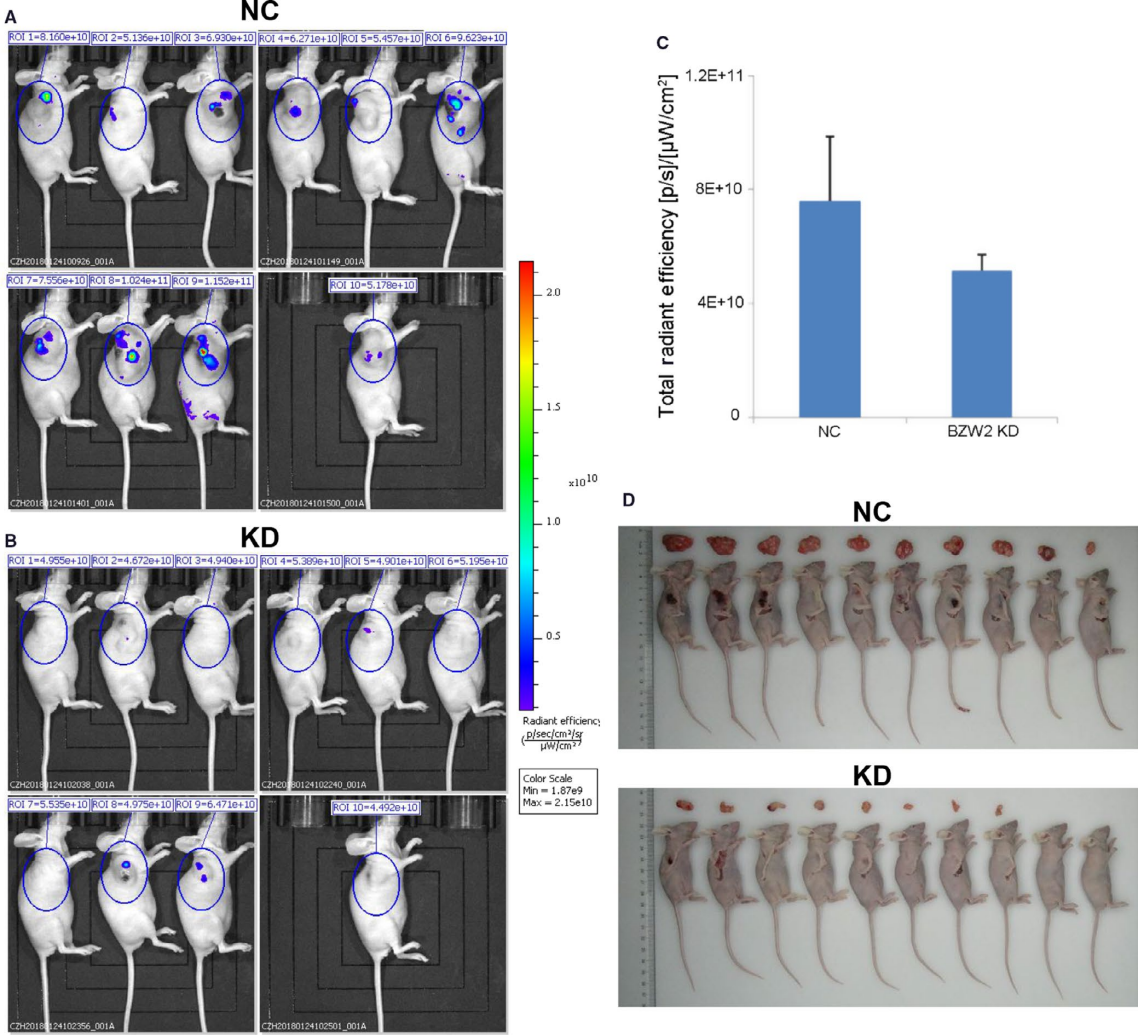
Representative bioluminescent imaging of tumours in human MIBC xenografts in mice, which were injected with T24 cells infected with the shRNA lentiviruses for the A, vector control (NC) or B, BZW2 gene (KD). C, Quantitative results of bioluminescent imaging. D, Resected tumours from mice bearing MIBC tumours after sacrifice (**P < 0.01)

Our results suggested that BZW2 could serve as a potential anti‐cancer target against MIBCs. To this end, we performed in vivo studies to assess the effect of BZW2 knockdown on the tumour growth in mice xenograft model. T24 cells infected with the shRNA lentiviruses for the BZW2 genes and vector control were injected into mice, followed by noninvasive bioluminescence imaging and tumour size measuring. From our results, it was obvious that BZW2‐knockdown significantly reduced the tumour volume in vivo (Figure 5A,B). The quantitative results showed that the total fluorescence expression in the region decreased from 7.61E+10 (NC) to 5.15E+10 (KD), the difference was statistically significant (*P* < 0.05) and the total radiation efficiency decreased around 32.3% (Figure 5C). The tumour proliferation was significantly inhibited in the BZW2 knock down mice. To confirm the imaging results, the mice were sacrificed to isolate the tumours, which size was further measured manually (Figure 5D). These findings strongly support the essential role of BZW2 in the tumorigenesis of MIBCs, which provides a solid basis for the target‐based therapies.

### 3.5 Microarray pathway analysis of signalling and disease states in response to BZW2 knockdown

**Figure 6:**
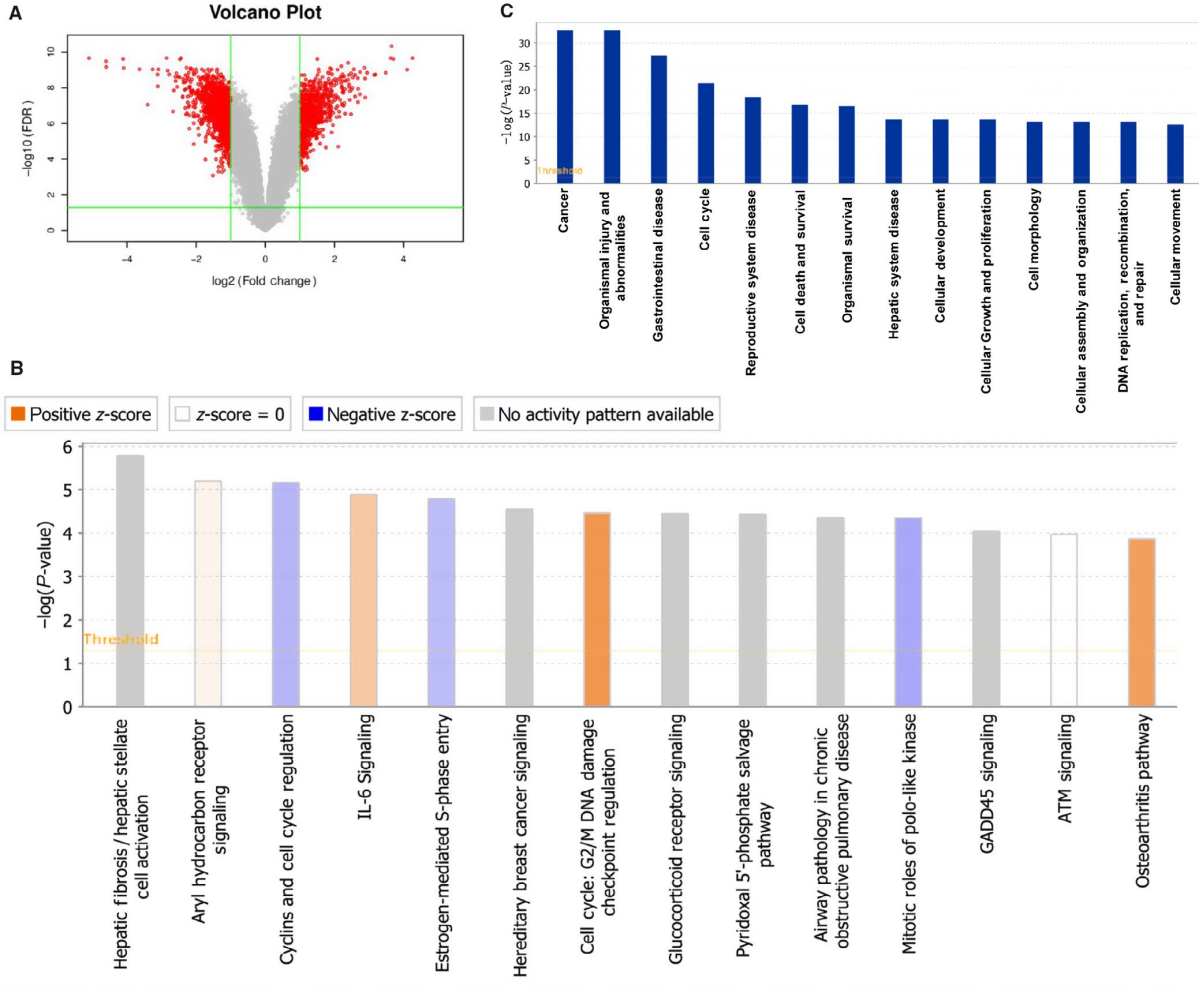
(A), Volcano plot showing proteins differentially expressed between the two groups (NC and KD). The up‐regulated and down‐regulated proteins are shown in red. Grey represents no significant change in expression level. B, Signalling pathways graph. The ratio represents differentially expressed genes from a pathway to the total number of genes of that particular pathway. A positive Z‐score represented an activated signalling pathway and disease functions, whereas a negative Z‐score represented an inhibited signalling pathway and disease functions. C, Significantly affected disease states and functions in response to BZW2 knockdown, as determined by sequencing of the –Log (*P*‐value)

To explore the possible molecular mechanism underlying the anti‐cancer effects of BZW2 in MIBCs, we performed microarray pathway analysis of the signalling and disease states which might be affected by the knockdown of BZW2. From our results, a total of 609 up‐regulated and 876 down‐regulated genes were identified in BZW2‐knockdown T24 cells compared with vector control cells. The volcano graph demonstrates the distribution of differentially expressed genes by fold change between the KD group and the NC group (Figure 6A).

On the above basis, we used IPA tools (http://www.ingenuity.com) to identify differentially expressed canonical pathways by defining the enrichment *P*‐value of the pathway. Signalling pathways associated with BZW2‐knockdown cells were sequenced by their ‐Log(*P*‐value) (Figure 6B)^23^ Notably, several pathways were found to be associated with cell cycle, including cyclins and cell cycle regulation,^24^ oestrogen‐mediated S‐phase entry,^25^ G2/M DNA damage checkpoint regulation,^26^ mitotic roles of PLK,^27^ ATM signalling.^28^ This is in agreement with the above experimental observations that BZW2‐knockdown induces G1‐phase cell cycle arrest in T24 cells. Besides, pathways associated with cancer and apoptosis were also significantly affected, eg hereditary breast cancer signalling, IL‐6 signalling, GADD45 signalling.^29^, ^30^ Other altered pathways include hepatic fibrosis/hepatic stellate cell activation, aryl hydrocarbon receptor signalling, glucocorticoid receptor signalling, pyridoxal 5′‐phosphate salvage pathway, airway pathology in chronic obstructive pulmonary disease and osteoarthritis pathway, which suggest the abnormalities in disease states and functions, eg biosynthesis.^31^

Furthermore, we evaluated the association of disease states and functions with biological networks on the basis of differentially expressed genes. The affected disease states and functions graph was presented on basis of the ‐Log (*P*‐value) (Figure 6C). As a result, 14 disease states and functions were significantly altered, including cancer, organismal injury and abnormalities, gastrointestinal disease, cell cycle, reproductive system disease, cell death and survival, organismal survival, hepatic system disease, cellular development, cellular growth and proliferation, cell morphology, cellular assembly and organization, DNA replication/ recombination/repair, cellular movement.

### 3.6 Gene associated with cell cycle regulation signalling pathways in response to BZW2 knockdown

**Figure 7:**
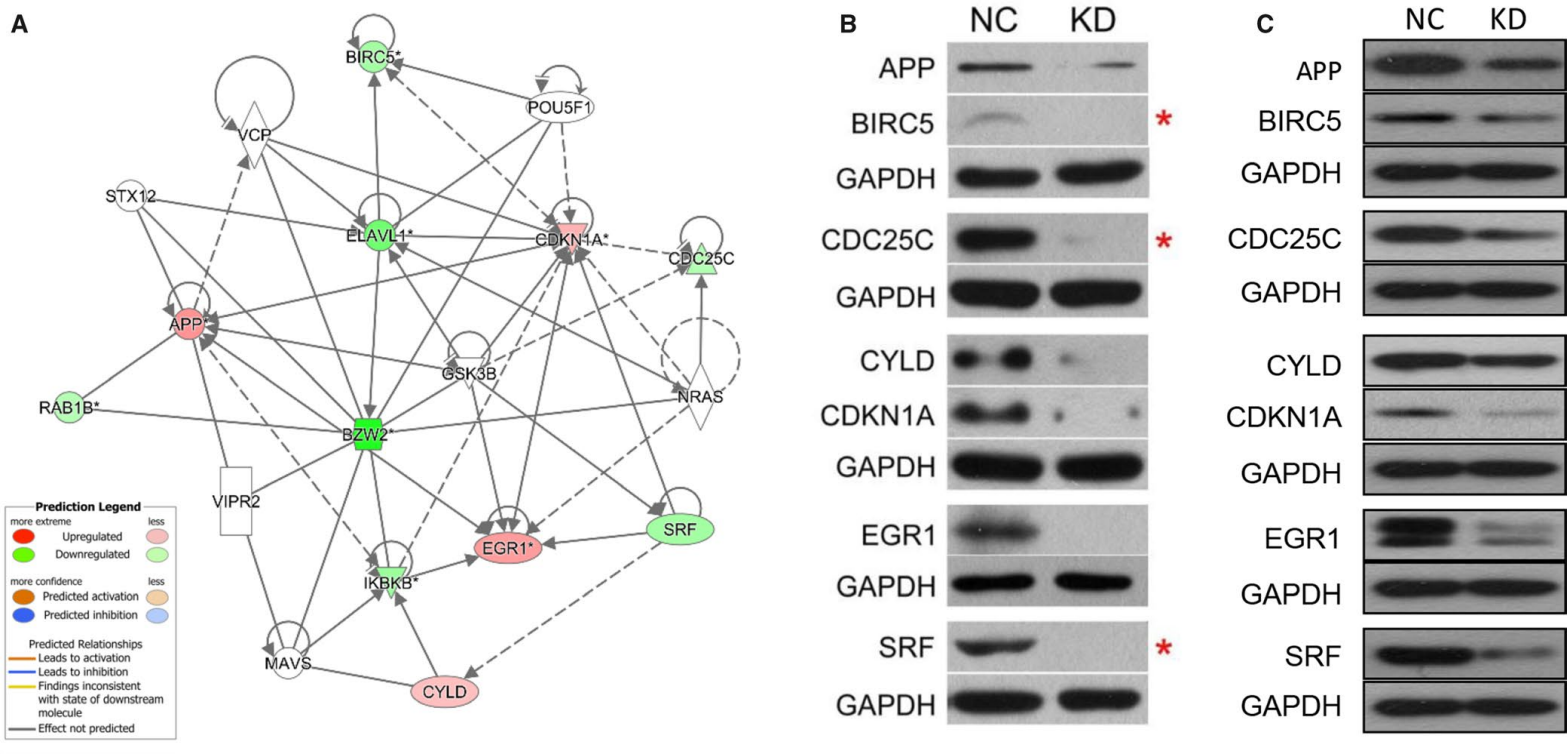
Alterations in cell cycle associated genes in response to BZW2 knockdown in T24 and 5637 cells. A, Network graph of the association between BZW2 and genes of cell cycle regulators. B, Results of Western blot analysis for selected targeted associated with cell cycle regulation in T24 and C, 5637 cells. GAPDH was used as a loading control

The network diagram of gene interaction analysed by IPA revealed interactions between BZW2 and molecules associated with the cell cycle regulation signalling pathways, including Survivin (BIRC5), CDC25C; CDKN1A (p21 or CIP1)^32^ Among them APP, EGR1, CYLD were upregulated, whereas BIRC5, CDC25C, SRF, ELAVL1, IKBKB, RAB1B were downregulated (Figure 7A). To confirm these results, we selected APP, BIRC5, CDC25C, CYLD, CDKN1A, EGR1 and SRF as the candidates for further investigation of the alternations upon BZW2 depletion in both T24 and 5637 cells with Western blot analysis. As expected, BZW2 knockdown significantly decreased the protein expression of BIRC5 (Survivin), CDC25C and SRF compared with that in the control cells (Figure 7B,C), which is consistent with the above results. To our surprise, there was significant reduction in the levels of APP, CYLD, CDKN1A and EGR1, which is different from the results of IPA analysis. Although it has been recognized that there is difference in the expression between mRNA and protein levels, further studies are in demand to explore the underlying mechanism.

### 3.7 The connection between the BZW2 and other genes of cell cycle regulators

**Figure 8:**
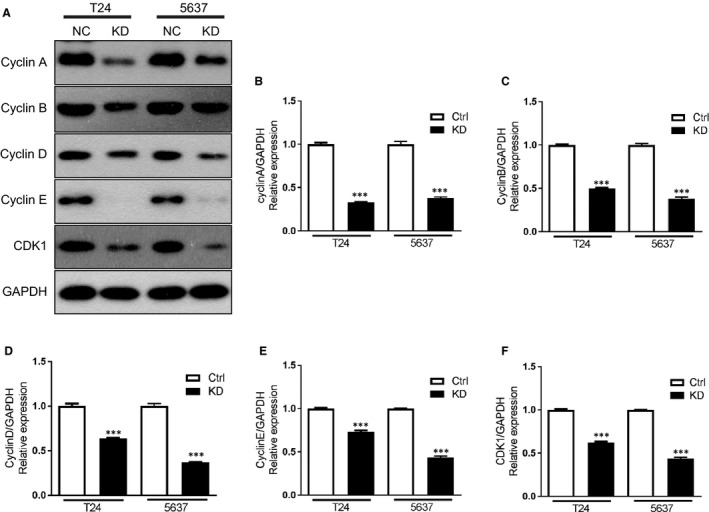
(A), The effects of BZW2 gene knockdown on the protein expressions of other cell cycle regulators genes. B, The effects of BZW2 gene knockdown on the mRNA expressions of Cyclin A, (C) Cyclin B, (D) Cyclin D, (E) Cyclin E and (F) CDKI genes in T24 and 5637 cells

For more in‐depth exploration, we crossed the differential genes with several well‐known genes involved in cell cycle regulation, and selected Cyclin A, Cyclin B, Cyclin D, Cyclin E and CDK1 genes to verify the link between BZW2 and other genes for cell cycle regulators. As expected, BZW2 knockdown significantly decreased the protein expression of above five genes compared with that in the control cells (Figure 8A). And we validated the above results from mRNA levels using q‐PCR analysis, which confirmed that the mRNA expression of the above five genes in BZW2‐knockdown T24 and 5637 cells was significantly lower than that of the control cells (*P* < 0.001) (Figure 8B‐F). These results indicated that BZW2‐knockdown may induce cell cycle arrest.

## 4 DISCUSSION

Muscle‐invasive bladder cancers (MIBCs) constitute approximately 20% of bladder cancer incidences, but account for the vast majority of cancer‐specific deaths, owing to the poor prognosis and more common progression to metastasis. To the best of knowledge, there is no reliable target or biomarker. Herein, we showed the expression of BZW2 is up‐regulated in the MIBC tissues as well as human MIBC cell lines. Given its potential role in osteosarcoma progression reported by Cheng et al,^11^ our results strongly suggested that BZW2 may have important functions in the tumorigenesis of MIBCs.

The knockdown of BZW2 gene in both 5637 and T24 cells consistently resulted in significant cell growth inhibition, reduction in viable cell number, cell cycle arrest at G1‐phase and apoptosis, indicating BZW2 could serve as a promising target for the treatment of MIBCs. Our results were consistent with previous report that almost all MIBCs have defects in genes encoding proteins that control the G1 cell cycle checkpoint.^9^ Despite the small percentage of incidences, MIBCs represent a great challenge in the clinic primarily owing to the vast majority of cancer‐specific deaths compared with other cancers. Moreover, the poor prognosis as well as the aggressive characteristics made currently available treatments far from satisfied. On these bases, there is an urgent demand for the new therapeutics and novel molecular target. On the other hand, improved treatment requires detailed understanding of MIBC pathogenesis and molecular biology. In this regard, our data provided a basis for further investigation.

The in vivo studies with xenograft mice models validated our assertion that BZW2 could serve as an effective anti‐cancer target against MIBCs, which was evidenced by the observations that BZW2 knockdown significantly inhibited the tumour growth. Our results gave the convincing supports and thus rationale for the future clinical or preclinical experiments gaining insights into the anti‐cancer potential of BZW2 with either small‐molecule drugs or small interfering RNA. To the best of knowledge, there is no such report illustrating the contributions of BZW2 in MIBCs with the combinations of both in vitro and in vivo studies.

Results from microarray analysis revealed the most altered signalling pathways and disease states, as well as the functions. Among them, cell cycle regulation associated pathways were enriched to the most, which involve a total of five signalling pathways, including cyclins and cell cycle regulation, oestrogen‐mediated S‐phase entry, G2/M DNA damage checkpoint regulation, mitotic roles of PLK, ATM signalling. This is in agreement with our experimental results that BZW2‐knockdown can induce G1‐phase cell cycle arrest. Notably, slightly different from 5637 cells, there is also accumulation of cells in G2/M phase observed in T24 cells, which might be explained by the up‐regulation of G2/M DNA damage checkpoint according to microarray analysis. As to the disease states and functions, cell cycle was also highlighted by the enrichment of differentially expressed genes. Besides, other identified cancer‐associated functions and states include cell death and survival, cellular development, cellular growth and proliferation, cell morphology, cellular assembly and organization and cellular movement, implying the important role of BZW2 in the tumorigenesis of MIBCs.

## CONFLICT OF INTEREST

All the authors have no conflict of interest to declare.

## AUTHOR CONTRIBUTION

Haifeng Gao: performed the research and drafted the manuscript; Guanghai Yu: conducted data curation and reviewed and edited the manuscript critically; Xian Zhang and Song Yu: analysed data and reviewed and edited the manuscript; Yu Sun and Yinghua Li: designed the research.
