# Supplementary figures and images for: BZW2 gene knockdown induces cell growth inhibition, G1 arrest and apoptosis in muscle‐invasive bladder cancers: A microarray pathway analysis
Source: J Cell Mol Med. 2019 Apr 1;23(6):3905–15. doi: 10.1111/jcmm.14266 (PMC6533564; doi:10.1111/jcmm.14266)

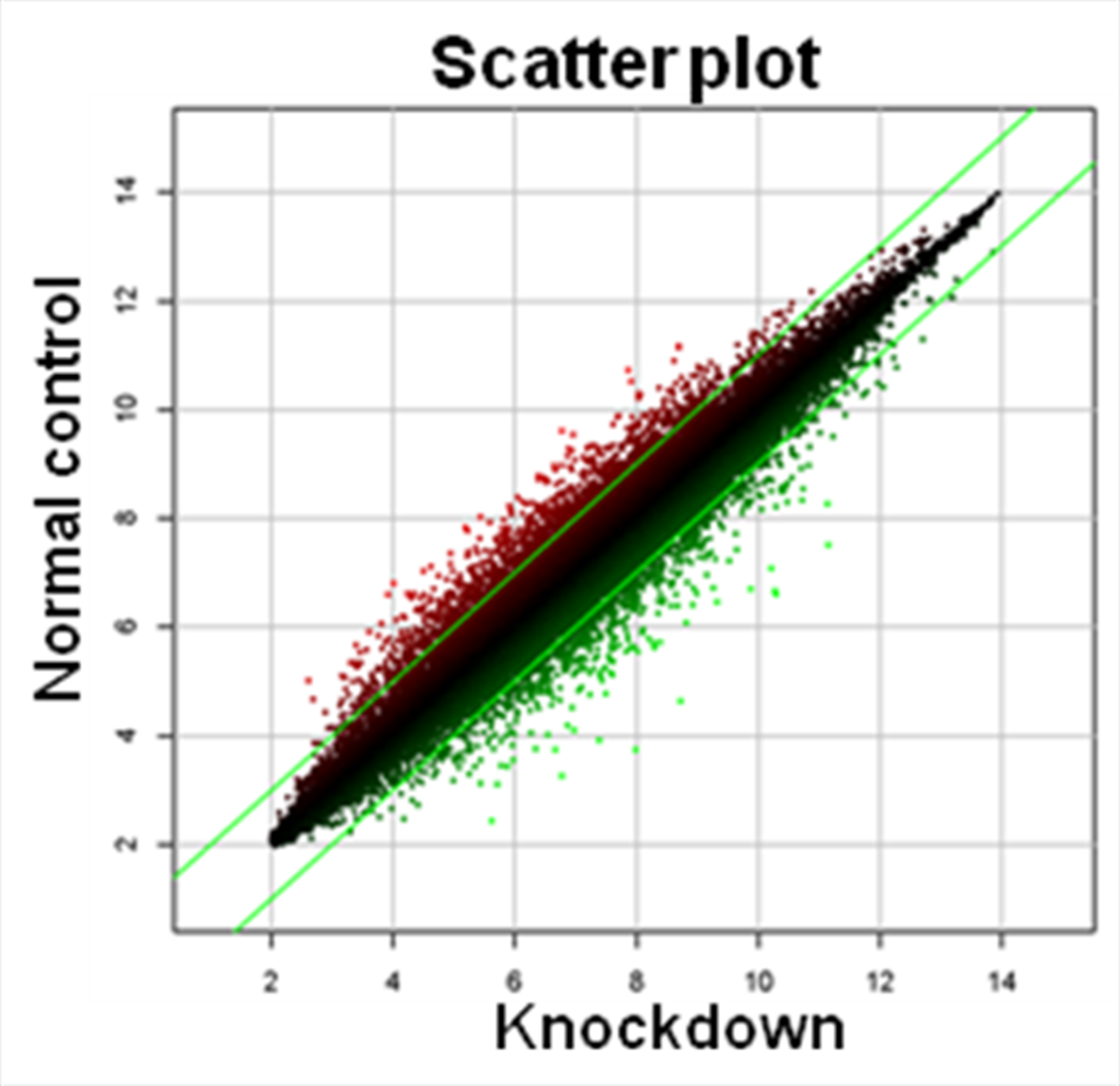

Supplement: Supplementary file 5 [file JCMM-23-3905-s005.tif]
